# Current Challenges and New Strategies in Pediatric Short Bowel Syndrome: Focus on Surgical Aspects and Prevention of Complications

**DOI:** 10.3390/children12050621

**Published:** 2025-05-12

**Authors:** Igor Sukhotnik, Haguy Kammar

**Affiliations:** Department of Pediatric Surgery, Dana Dwek Children’s Hospital Tel Aviv Sourasky Medical Center, 6 Weizmann St., Tel Aviv 6423906, Israel

**Keywords:** short bowel syndrome, children, surgery, lengthening procedure, serial transverse enteroplasty, longitudinal intestinal lengthening, parenteral nutrition

## Abstract

**Background:** The medical management and non-transplant surgical options for children with short bowel syndrome (SBS)are maximized as first-line treatments. The purpose of this review is to summarize the currently available evidence and new management strategies in children with SBS. **Methods:** A systematic review of the literature was conducted on data from the last four years, focusing on both the effectiveness and safety of intestinal lengthening procedures, as well as frameworks for the prevention of complications and the achievement of enteral autonomy. **Results:** Of 546 abstracts that were screened, a total of 27 relevant full-text articles published between 2021 and 2025 were reviewed. The literature that was review showed that, over the past four years, the most commonly used lengthening procedure was serial transverse enteroplasty (STEP), which resulted in a 50–70% increase in bowel length, a decrease in PN dependency in most cases, and weaning off PN in 42–73% of patients. The longitudinal intestinal lengthening technique (LILT) has been used less frequently, allowing a similar 70% increase in small bowel length and 32–52% of patients to wean off PN, but with a higher mortality rate. The main reasons for surgery in patients with SBS patients were the inability to wean off PN, intestinal dysmotility, and bacterial overgrowth. Over the last decade, several new techniques—such as induced intestinal lengthening, distraction enterogenesis, ileal lengthening through internal distraction, and double-barrel enteroplasty—have been described as options for the treatment of a limited bowel length and less invasive modalities. **Conclusions**: Autologous gastrointestinal reconstructive surgery, as a part of multidisciplinary management, remains vital for managing children with SBS.

## 1. Introduction

In 2016, the European Society for Clinical Nutrition and Metabolism (ESPEN) published an updated version of guidelines that defined intestinal failure (IF) as “the reduction of the gut function below the minimum necessary for the absorption of macronutrients and/or water and electrolytes, such that intravenous supplementation is required to maintain health and/or growth” [1]. In the pediatric population, IF is defined as a decrease in the actual functional intestinal mass below that which can sustain normal growth, satisfactory nutrient absorption, or a satisfactory electrolyte balance, resulting in dependence upon parenteral nutrition (PN) for a minimum of 60 days within a 74 consecutive day interval [2]. Short bowel syndrome (SBS) is one of the most frequent mechanisms of IF and can result in a loss of intestinal length following surgery, congenital anomalies of the gastrointestinal tract (GIT), and a variety of intestinal pathologies or damage [3,4]. In adults, SBS is defined by the ESPEN as IF associated with a residual small bowel shorter than 200 cm [1]; there is no consensus regarding SBS definition in children. Although the intestinal remnant length is commonly used to define pediatric SBS, many centers consider SBS as being a functional rather than an anatomic entity. The Canadian Association of Pediatric Surgeons defines SBS as the need for PN for longer than six weeks after massive gut resection, or a residual intestinal length of less than 25% of that which is expected for the patient’s gestational age [4].

SBS is categorized into three types based on the residual intestine anatomy: type 1—SBS with end jejunostomy/ileostomy; type 2—SBS with jejuno colic anastomosis; type 3—SBS with jejunoileal anastomosis, an intact colon, and an ileocecal valve [1]. The clinical features of SBS are characterized by diarrhea, dehydration, electrolyte imbalance, malabsorption, steatorrhea, progressive malnutrition, and requiring long-term PN [3,4]. The management of SBS primarily focuses on intestinal rehabilitation using a multidisciplinary approach that includes nutritional, medical, and surgical interventions to enhance intestinal adaptation, improve the function of the residual bowel, and achieve enteral autonomy [5]. PN is a life-saving treatment for patients with SBS, and the prognosis of SBS has greatly improved during the past decades because of its availability [4]. Supplemental PN is required to maintain the levels of fluid, electrolytes, trace elements, and vitamins, as well as the nutrient balances, in children with SBS and IF; however, the long-term use of PN is associated with many complications, including catheter-related bloodstream infections, intestinal failure-associated liver disease, catheter-related vascular thrombosis, metabolic abnormalities, small intestinal bacterial overgrowth, metabolic bone disease, and renal impairment. One of the primary goals of SBS therapy is to reduce or eliminate dependence on PN and to increase the probability of successful PN weaning. Limiting the duration of PN by promoting enteral autonomy (EA) and the optimization of the patient’s enteral nutrition has been shown to decrease complications and improve survival for pediatric patients with SBS and IF [6]. We aim to achieve EA before life-threatening complications related to the chronic use of PN develop [7]. Although the radical treatment of patients with SBS consists of intestinal transplantation, this procedure is still associated with a high risk of severe complications such as infection, rejection, and other complications related to immunosuppression. Therefore, the medical management and non-transplant surgical options should be maximized as first-line treatments. The primary objectives of surgical procedures for children with short bowel syndrome (SBS) are as follows:

(1) Slowing intestinal transit time: this is aimed at patients who experience the rapid passage of chyme through a short bowel remnant with effective propulsive peristalsis and without bowel dilation;
(2) Improving intestinal motility: in cases where the bowel is dilated, leading to abdominal distention, food intolerance, bilious vomiting, bacterial overgrowth, translocation, and lactic acidosis, this involves reducing the bowel diameter and restoring normal lumen function;
(3) Increasing intestinal length: for children with a short bowel remnant and limited mucosal surface area, this procedure aims to enhance long-term enteral nutrition prospects and reduce dependency on parenteral nutrition (PN) when weaning from PN progresses too slowly;
(4) Increasing intestinal absorptive surface area [8,9].

Increasing the intestinal transit time in children with SBS allows sufficient time for digestion and absorption. Additionally, increasing the length of the contact time of intraluminal nutrients with intestinal mucosa stimulates intestinal adaptation and regrowth. A variety of surgical procedures have been developed to reduce the intestinal transit time. These include antiperistaltic intestinal segment, surgery on intestinal valves and sphincters, and isoperistaltic interposition of the colon. These techniques are only indicated in patients with good propulsion of luminal nutrients and are contraindicated in cases of intestinal dysmotility. For those children with SBS who developed intestinal dysmotility with inefficient peristalsis and dilated intestinal loops, several reconstructive surgical techniques have been developed. These include intestinal tapering, the longitudinal intestinal lengthening technique (LILT procedure), serial transverse enteroplasty (STEP procedure), and spiral intestinal lengthening and tailoring (SILT).

The objective of this report is to present a current review of the literature regarding the efficacy and safety of intestinal lengthening procedures in pediatric patients with short bowel syndrome (SBS). Additionally, this report aims to outline strategies for preventing complications and achieving enteral autonomy. The following sections discuss indications, advantages, and disadvantages of the surgical techniques and complications, as well as addressing controversial questions, regarding lengthening procedures in children with SBS, and also addresses recent experimental research in the last four years.

## 2. Materials and Methods

**Figure 1:**
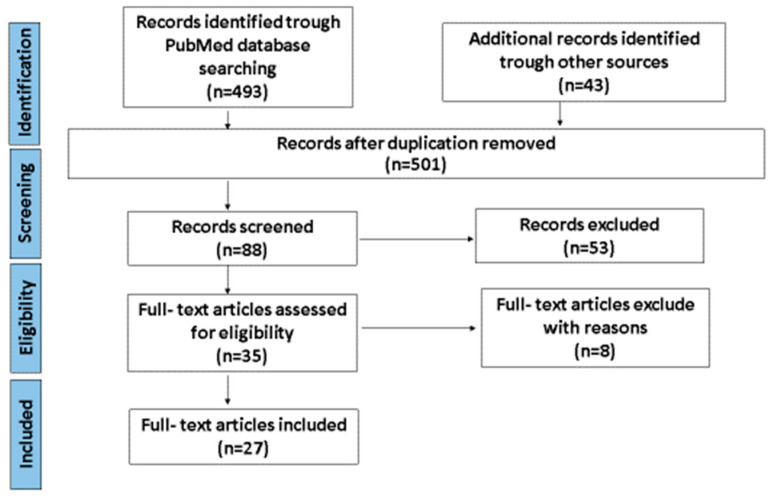
PRISMA diagram of the selection process of included studies. Eighty-eight studies underwent full-text review, of which 53 studies met inclusion criteria and had extractable data. The other eight studies were excluded for the following reasons. Finally, 27 full-text articles were included.

A comprehensive literature search was performed using the data published in the MEDLINE-PubMed Scopus, Web of Science, Cochrane Central Register of Controlled Trials (CENTRAL), Scientific Electronic Library Online (SciELO), and Google Scholar databases between January 2021 and December 2024, and through a manual search of the reference lists of relevant studies. The search terms included various combinations of keywords such as “short bowel syndrome”, “intestinal failure”, “children”, “surgery”, “complications”, “lengthening procedure”, “transit time”, “STEP”, “LILT”, “antiperistaltic intestinal segment”, “intestinal valves and sphincters,”, “isoperistaltic interposition of colon“, “stoma”, and “gastrostomy”. Eligibility criteria were based on the PICO (population, interventions, comparators, and outcomes) elements of the review question, and a specification of the types of studies that have addressed these questions (Table 1). Two authors independently screened the titles and abstracts of the initial search results. Full-text papers, including analytical cross-sectional studies, prospective cohort studies, case control studies, longitudinal studies, randomized controlled trials, case series, and retrospective cross-sectional studies, were included. Studies that did not meet the research objectives, as well as non-English publications, editorials, letters to the editor, articles without freely available abstracts, duplicate entries, abstracts, and conference proceedings, were excluded from the review. A flowchart with a schematic representation of the selected articles is provided in Figure 1. The STROBE Statement checklist of cohort, case-control, and cross-sectional studies (combined) [10] and the JBI checklist for systematic reviews and meta-analyses [11] have been used to assess the methodological quality of the studies. The researchers complied with the Preferred Reporting Items for Systematic Reviews and Meta-Analyses Extension for Scoping Reviews (PRISMA-ScR) guidelines, which informed decision-making, data extraction, and reporting.

The study protocol was registered in the PROSPERO database (ID 1023895). There were no important deviations from the protocol. The authors used the Cochrane Risk of Bias 2.0 tool [12] to assess bias in the retrieved studies, with judgments falling in the categories of “high risk of bias”, “low risk of bias”, or “some concerns”. Another senior researcher intervened when an agreement was not reached. In parallel, the quality of the included studies was evaluated by both reviewers using the COSMIN scoring system to determine the quality ratings on a 4-point scale (e.g., poor = 0, fair = 1, good = 2, and excellent = 3) [13].

## 3. Results and Discussion

### 3.1. General

A total of 2410 unique titles were retrieved from the literature search. After a review of the title, abstract, and publication year of the articles, 1864 articles were excluded. Between 2021 and 2025, a total of 546 articles were identified in the field of short bowel syndrome in children: MEDLINE-PubMed Scopus—493 articles, Web of Science—36 articles, Cochrane Central Register of Controlled Trials (CENTRAL)—0 articles, Scientific Electronic Library Online (SciELO)—7 articles, and Google Scholar—10 articles. Eighty-eight records were screened, and of them 53 records were excluded. Following full text review of the remaining 35 articles, 8 articles were excluded for different reasons. Finally, 27 full-text articles were reviewed. In total, 15 studies discussed the efficacy of STEP, 4 studies reported on LILT and compared LILT to STEP, 2 studies examined the effects of a re-STEP, and 5 articles discussed new techniques and combined procedures. In all studies, the quality of reporting papers was considered to be from poor to fair. The primary reasons for the low quality scores among these papers were the small sample size and the absence of proper statistical analysis. Additionally, as most studies were of low methodological quality and all were retrospective series, firm conclusions cannot be drawn due to the potential for reporting bias.

### 3.2. Bowel Lengthening Procedures

Autologous gastrointestinal reconstructive surgery (AIRS) is an important tool in the multidisciplinary management of children with SBS. Once a patient with SBS has reached the plateau of intestinal adaptation despite receiving optimal medical and nutritional treatments, surgical routes may be considered: AIRS and small bowel transplantation. The most commonly used AIRS procedures are STEP and LILT. In a recent review article, Nagelkerke et al. systematically reviewed the evidence on the outcomes of two surgical techniques—STEP and LILT in children with SBS. Forty papers were included in this review, discussing 782 patients [14]. This review, unlike the previously published one, did not include study selection and case reports. Data extraction was conducted by two independent reviewers. An analysis of the patients revealed that 46% of children with SBS were weaned off PN after STEP and 52% were weaned off it after LILT. The mortality was 7% for STEP and 26% for LILT. The opposite was observed by two studies comparing LILT to STEP procedures that found no difference in survival between the groups [15,16]. The percentage of PN decrease was 52% for STEP and 66% for LILT, which alignes with previous findings [15,16]. The most common indications for the STEP procedure were failure to advance on enteral feeds (77%), bacterial overgrowth (13%), and intestinal atresia (10%). The indication for the LILT procedure (201 patients) was a failure to advance on enteral feeds for a 6-month period of maximum conservative treatment. The median percentage of bowel length increase was 49.5% after STEP (could not be calculated for LILT operation). Two years later, the same group has made a separate analysis excluding all studies from similar medical centers with complete overlap with regard to the time period studied. Eleven studies discussed the effects of the first LILT (213 patients) and 11 studies discussed the effects of the first STEP (279 patients). The authors have reported that 56% of patients after a LILT procedure and 54% of patients after a STEP procedure achieved enteral autonomy at the end of follow-up. However, the reported mortality rate at the end of follow-up was higher after the LILT procedure (21%) compared to the STEP operation (6%). Similar to their previous review paper [14], the authors conclude that LILT and STEP are both valuable treatment strategies for pediatric SBS. However, it is not possible to advise surgeons on accurate patient selection or to predict the result of either intervention [17]. Tsang et al. [18] reported the experiences and outcomes of 64 children with SBS (median bowel length was 45 cm) managed by a multidisciplinary intestinal rehabilitation program in a tertiary pediatric center over two decades [18]. The authors note an 89% survival rate and a 78% enteral autonomy rate over the mean follow-up period of 8.9 years, which is significantly higher than the previously published reports [14,17]. Among 50 patients who weaned off PN, two patients underwent bowel lengthening procedures.

### 3.3. Serial Transverse Enteroplasty Procedure (STEP) Procedure

The serial transverse enteroplasty procedure technique (STEP) was first described by Kim et al. in 2003 [19] in a pig model, and the first clinical application of this procedure was reported later in the same year [20]. STEP is currently considered the most common small bowel lengthening procedure worldwide [21]. The anatomical principle of STEP considers that the intestinal blood supply originates from the gut mesenteric margin and flows perpendicular to its longitudinal axis.

**Figure 2:**
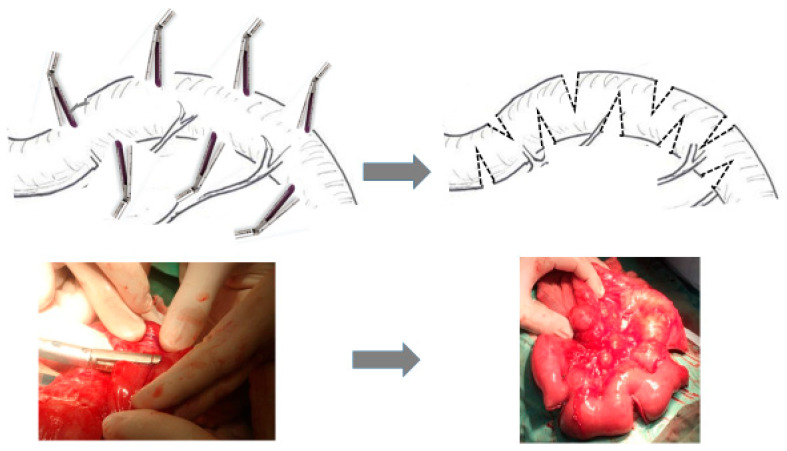
In STEP procedures, staplers are fired from alternative sides perpendicular to the direction of the bowel with zig-zag pattern is achieved. Photograph showing the small bowel after STEP.

The STEP procedure (Figure 2) involves making alternating cuts with a GIA™ linear stapler from opposite directions on the dilated intestine, perpendicular to the long axis. This results in a zig-zag pattern, decreasing the bowel width and increasing its length while maintaining parallel blood flow to the suture lines for adequate vascularization.

Since 2007, a “STEP Registry” has been established to systematically gather comprehensive data regarding the outcomes of the procedure. The registry aims to identify indications of, evaluate the efficacy of, and assess complications associated with STEP, as well as to establish criteria for patient selection and the optimal timing of interventions [22]. The most common indications include dependence on parenteral nutrition, bacterial overgrowth in the context of short bowel syndrome, and neonatal atresia with a marginal residual bowel length. Overall, the mean intestinal length increased considerably by 69% with a relative three-fold tapering of the dilated intestinal diameter. Short-term complications of STEP include staple-line leaks, postoperative bleeding, and intestinal obstruction. Long-term complications are infectious complication, chronic gastrointestinal bleeding from ulcerations at staple sites, and re-dilatation of the small bowel.

In 2013, Jones et al. reported data on 111 consecutive patients who were enrolled in the International Serial Transverse Enteroplasty (STEP) Data Registry. Forty-seven percent of patients achieved enteral autonomy after the first STEP, with a better chance being observed for patients with a longer pre-STEP bowel length. The median time to reach enteral autonomy was 21 months. Overall, the mortality post-STEP was 11% [23].

In the past four years, 15 published papers have discussed outcomes of STEP: 6 review papers, 5 retrospective cohort studies, 1 case report, 2 comparative studies (STEP and re-STEP), and one STEP modification. In five retrospective studies, a total number of 93 patients were described, with a median of 18 patients per study (range: 6–36). The main aims of the discussions were the best timing for STEP procedures, the main indications for STEP, and the effectiveness of STEP (Table 2).

Lauro et al. [25] recently completed surveys on STEP which were published in the international literature from 2003 to 2021, including 23 comparative and cohort studies from 17 countries. STEP was performed on 308 children and adults. The pediatric group included 16 studies. The post-STEP increase in small bowel length for children ranged between 42 and 100% and enteral autonomy was reached in 32.22% of cases. The frequency of PN dependence was 36.11% and the mortality rates were around 5.55%. A repeated STEP procedure was needed in 17.22% of children. The authors report that a preoperative length with preservation of the ileocecal valve represented the main predictive factor of achieving enteral autonomy [20]. This review paper did not provide any new insights and many of the studies summarized have already been included in earlier reviews. In a recent study, Dagorno et al. [24] performed the first French multicenter national retrospective study, reporting the long-term outcomes of the STEP procedure in 36 children with SBS between 2000 and 2022 in six University Hospitals, with a median follow-up of 7 years. Consistent with the first report from the STEP Registry [22], the main indications for STEP procedures were bacterial overgrowth, impossibility to wean from PN, and intestinal atresia with SBS. The median age at first STEP was 10.8 months and the median remaining bowel length was 47 cm. Following the operation, the bowel length significantly increased (50%), with a median gain of 16 cm. Fourteen (42%) children were weaned off PN. In the remaining 19 children, the PN dependency decreased by 19%. These findings agreed with previously published data [20,21,22,23,25]. However, other studies have reported higher rates of enteral autonomy after STEP, ranging from 60 to 91% [31,32]. Seventy percent of children who had been operated on for severe intestinal dilations and bacterial overgrowth were completely asymptomatic post-surgery. The authors conclude that the STEP procedure remains a good surgical option in the management of children with SBS, especially for children with intestinal dysmotility, allowing for decreased PN dependency in most cases and allowing for weaning off of PN in some cases. Cardo Almeida et al. described the outcomes after autologous gastrointestinal reconstructive surgery (AGIR) in 27 patients from two Centers in the UK. Twenty patients underwent the STEP procedure, which resulted in an increase in small bowel length of 70%. These findings were consistent with a systemic review comparing outcomes of STEP vs. those of LILT that indicates a comparable 70% increase in small bowel length between the two methods [31]. Thirty-five percent of patients with 25% of the expected small bowel length achieved enteral autonomy, which is in agreement with recent French study [24] but lower compared to other reports [32,33]. The authors consider that significant differences in the rates of enteral autonomy between groups are likely to be multifactorial, including technique-related aspects, the patient selection, the etiology of the SBS, and the stage of intestinal adaptation. The authors conclude that AGIR is an important tool in the multidisciplinary management of children with SBS and that the percentage of estimated small bowel length and the etiology of the SBS are likely predictors of achievement of EA in patients undergoing AGIR [26]. Recently, our group investigated the clinical features and complications of intestinal dysmotility in children with IF and SBS [27]. The STEP procedure was performed in 11 SBS-dysmotility patients (61%) and the requirement for the STEP procedure was found to be significantly lower in patients with SBS without dysmotility. In all patients, STEP “saved” their dysfunctional intestine. Eight of these patients (73%) were weaned from total parenteral nutrition (TPN). This is significantly higher than the 47% reported by the international STEP Registry; this number was based on 111 patients [23]. We conclude that the successful treatment of infants with SBS with intestinal dysmotility may be achieved using STEP. Velasquez et al. describe a case of concomitant jejunal atresia and Hirschsprung’s disease that resulted in SBS and PN dependence for five years. The authors discuss the possibility of using STEP to manage the dilated proximal bowel and gain length followed by subtotal colectomy and ileoanal pull-through. The authors conclude that STEP can be successfully utilized in patients with a history of Hirschsprung’s disease and jejunal atresia to achieve nutritional autonomy and ultimately reestablish gastrointestinal continuity with pull-through [34]. Bueno et al. recently described a technical modification of the STEP procedure (MSTEP) that consists in stapler application without mesenteric defects, and this method can also be applied to the duodenum. Sixteen children with SBS were operated on using this technique. Forty-five percent of patients achieved enteral autonomy (four patients after the first operation and three patients after the redo procedure). The authors conclude that the effectiveness of MSTEP in achieving enteral autonomy seems similar to the classical STEP [20,21,22,23,25] and that the retained colon length may influence the achievement of post-STEP enteral autonomy [28]. The advantage of this technique is that it can be applied to the duodenum. Schmedding et al. conducted a retrospective review of 435 neonates (using data from a major health insurance company, which cover ~30% of the German population) with jejunoileal atresia who were treated during a 10-year period between 2007 and 2016. The median follow-up was 4.6 years. SBS was coded in 22.3% of patients, but only 1.8% of all children required intestinal lengthening. Six children (1.4%) underwent the STEPS procedure, two of them twice and one three times, and another two children required LILT. The presented data are similar to the data reported in the literature [29]. Lemoine et al. analyzed the data of all STEP procedures done at their institution (freestanding pediatric hospital) between June 2004 and December 2019. Twenty-four patients underwent 32 STEP procedures (16 oneSTEP, 8 re-STEP). Feeding intolerance was the most frequent indication in all patients after their first STEP (71%), while the indications for re-STEP were equally distributed between feeding intolerance and infections. The mean increase in bowel length was 60%. Thirty-seven-point-five percent (9/24) of patients achieved enteral autonomy at last follow-up: 7/16 after one STEP, 2/8 after reSTEP. These data align with another systemic review regarding STEP and LILT [31] and a recent French trial [31]. The authors conclude that similar postoperative outcomes after reSTEP and single-STEP (improved enteral tolerance, reduced rates of infections) support the use of reSTEP when it is clinically indicated, although reSTEP in young infants with a history of gastroschisis may need further evaluation [35]. In a recent retrospective comparative study, Mercer et al. performed a retrospective review of their experience (From 1 January 2008 to 1 January 2018) with a second STEP procedure in 23 patients with SBS. The median age at the time of second STEP was 2.2 years. The median operative time was 135 min with estimated blood loss of 5 mL. The median bowel length was 68 cm before and 85 cm after the second STEP (25% increase). The median time to resumption of enteral feeds was 7 days. Excluding one child who never received PN at all, 12/22 children were completely weaned off PN and 10 remained on at least some support. The authors conclude that a second STEP procedure provides additive benefit to most children presenting with bowel dilation following a prior STEP procedure and when PN cannot be weaned off of despite maximal medical therapy [30]. Nhan et al. reported two cases of congenital short bowel syndrome in the same family. This rare neonatal gastrointestinal disorder is characterized by a congenital, significantly reduced length of the small intestine. Although surgical interventions (Ladd’s procedure, gastrostomy, central line insertion, etc.) are a part of the multifaceted strategy for patients with this congenital anomaly, bowel lengthening procedures have not been recommended for these infants [36].

### 3.4. LILT, Comparison with STEP

**Figure 3:**
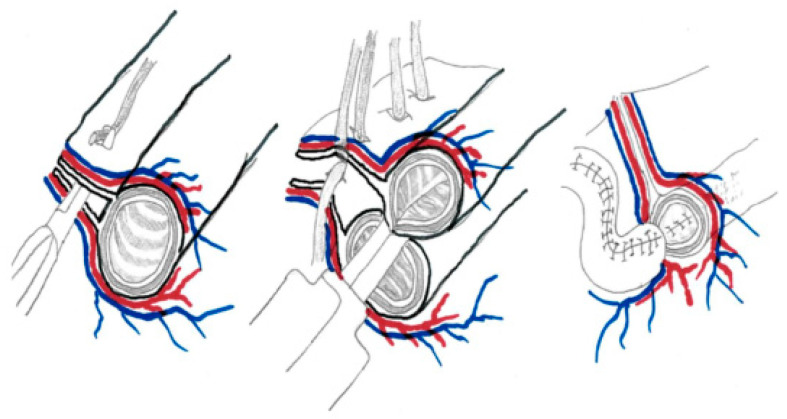
In LILT procedures, the small bowel is divided longitudinally in the midline along the mesenteric and antimesenteric border after separation of the mesentery vessels to the left and right side.

The longitudinal intestinal lengthening technique (LILT procedure) was first described by Bianchi in 1980 in a pig model [37] and was initiated in clinical practice by Boeckman and Taylor in 1981 on a 4-year-old boy with SBS (secondary to gastroschisis). A 30 cm long section of distal small bowel remnant underwent the LILT procedure and was anastomosed with the transverse colon. After 10 weeks, the patient reached full enteral autonomy, and parenteral support was stopped [38]. The indications for this procedure were dependent on PN and the impossibility of achieving at least 50% of the patient’s caloric requirement enterally after six months of adequate conservative treatment [39]. The LILT technique was designed to double the length of a loop of dilated small intestine and is based on the anatomical peculiarity of mesenteric vessels entering the bowel from either side of the midline, with anterior and posterior branches providing blood supply to each half of the circumference of dilated bowel segments that are allocated alternately to one or the other side of the bowel loop (Figure 3). Therefore, there is a larger avascular space between the two vessel layers. During this procedure, the small bowel is divided longitudinally in the midline along the mesenteric and antimesenteric border after separation of the mesentery vessels to the left and right side. The procedure requires a minimal intestinal diameter ≥4 cm or at least a dilatation of double the normal SB size and a healthy mesentery. The dilated segment of the small bowel should be longer than 20 cm. Such length is obligatory to avoid volvulus of the created bowel loops after longitudinal dissection and anastomosis.

The main disadvantages of the LILT procedure include the obligatory presence of healthy nonfibrotic mesentery with good vascular configuration; a fixed degree of tailoring and the ability to only reduce the diameter to half and double the length; and the inability to perform redo-procedure on the same segment while it is feasible after prior STEP. The manipulation and dissection of the mesentery is significant, as it increases the chance of vascular compromise and subsequent necrosis of one of the hemi-loops. The main outcomes of the LILT procedure are presented in Table 3. Studies show PN weaning rates between 32% and 52%, an average bowel length increase of 42–70%, a decrease in PN dependency by 19–36%, and mortality rates from 4% to 22%. LILT complications may include ischemia, suture leakage, abdominal abscesses, small bowel stenosis, intestinal fistulas, and blind intestinal loop formation [37,38,39].

LILT and STEP are the two principal procedures used to lengthen the native bowel in children with a SBS. However, the indications, the best timing for performing the lengthening procedure, and their outcomes remain controversial. The choice between performing a STEP or a LILT is often down to surgical expertise and preference; however, the intestinal anatomy often plays an important role in the decision making. In 2013, Frongia et al. reviewed 39 publications comparing the LILT and STEP procedures in children with SBS. The main indication for lengthening was the failure to achieve intestinal autonomy by conservative therapy, and end-stage liver disease was reported as the main contraindication. Although a sufficiently dilated intestinal segment was a common anatomical precondition for both procedures, STEP can be performed on shorter intestinal segments and on intricate segments such as the small bowel dilatation in ultra-short bowel syndrome and duodenum, which is technically not feasible for LILT. Both procedures have a similar extent of intestinal lengthening (approximately 70%) and result in improvement of enteral nutrition and the reversal of complications of PN. STEP seems to have a lower mortality and overall progression to transplantation. LILT has proven its value in AGIR, but its availability depends on the expertise of the surgeons [31]. A recent review (on behalf of the Italian Society of Gastroenterology, Hepatology and Nutrition) aimed to investigate the role of lengthening procedures in achieving enteral autonomy. This group conducted a systematic literature search to identify studies published from January 1999 to 2019, resulting in the identification of 947 patients. The authors have reported that the prevalence of weaning off PN was significantly higher in patients treated with PN alone (61.6%) than in patients receiving any AGIR (46.2%). The patients receiving AGIR had greater survival rates compared to only-PN patients (95% vs. 91.5%, NS) and the incidence of liver disease was significantly higher in the patients treated with PN alone than in the patients treated with AGIR. One limitation of this review is that the two groups are not fully comparable. Because an indication for AGIR is an impossibility of increasing the enteral tolerance, patients receiving AGIR could have had more severe IF, even if the mean intestinal length was comparable in the two groups. The authors concluded that AGIR may be useful in selected patients [40]. The STEP and LILT procedures elongated the bowel up to 75% and 100%, respectively. The chance to be weaned off PN following the STEP and LILT procedures ranged from 6% to 67% and from 55.5% to 100%, respectively.

In the last 4 years, only four papers have discussed the outcomes of the LILT procedure, suggesting a decreasing popularity of this technique in the last decade. No articles were found that discussed this operation exclusively. Three articles reviewed data from the literature regarding LILT and two retrospective case-series articles compared the LILT and STEP procedures (Table 3).

Kießling et al. studied the effects of massive bowel resection and various treatments on the quality of life in children and adolescents with SBS. The study included 57 children: 9 underwent LILT, 3 underwent STEP, and 2 received both procedures. They found that patients who underwent LILT (Bianchi) had a significantly higher average quality of life than those who had STEP. However, due to small sample sizes and other limitations, these results cannot be generalized to all patients with SBS [41]. Cardoso Almeida compared the outcomes of AGIR procedures (STEP—20 procedures vs LILT—9 procedures) in 27 patients from two UK centers. The pre-operative bowel length and age at surgery were similar for both groups. No significant difference was found between STEP and LILT. LILT increased the small bowel length by 70%, and the rate of weaning off PN was 44%, aligning with other reports [39,40,41].

### 3.5. Timing and Main Indications for Lengthening Procedures

The optimal timing and primary indications for lengthening procedures in infants and children with SBS are still debated. According to a review by Nagelkerke et al., the median age at the first STEP among 377 patients was 12 months. The median age at reSTEP was 24 months, and the median age at LILT was 22 months [14]. In the series by Lemoine [35], the age at first STEP was 2.37 years and, at one-STEP, patients were 3 years old. In the series by Mercer et al. [30], the median age for the entire cohort at first STEP was 6 months and the median age at the time of second STEP was 2.2 years. In a recent study, Zulli et al. reported their institutional experience of bowel lengthening procedures performed in 10 infants before 6 months of age. The median age at surgery was four months. Indications for early lengthening were re-dilatation after primary anastomosis and failure of enteral nutrition. The survival rate was 90%. Four children were weaned off PN and four needed partial home PN. After reviewing the data from the literature, the authors note that their data fit with those of other authors who recommended a lengthening procedure only after a period of bowel adaptation, since the natural adaptive process may obviate the need for any other interventions. The authors believed that early lengthening procedures should be considered only in cases of actual necessity [42]. The indications for lengthening procedures also remain a controversial issue. In accordance with Hosie et al. [40], an indication for a lengthening procedure is the impossibility of achieving at least 50% of the patient’s caloric requirement by enteral nutrition after 6 months of adequate conservative treatment. Other indications have also been discussed. In the series by Bueno et al. [28], indications for the STEP procedure were nutritional autonomy achievement (n = 11) and bacterial overgrowth treatment (n = 5). In accordance with a recent French study [20], the main reasons for surgery were an impossibility to wean off of PN (18 patients), bacterial overgrowth with major dilations (15 patients), and SBS due to intestinal atresia (3 patients). Many children with SBS underwent a STEP procedure due to severe intestinal dysmotility resulting in chronic obstructive symptoms or major intestinal dilatations that affected their condition. In a recent study by Lemoine et al. [35], feeding intolerance (a symptom that is usually related to intestinal dysmotility) was the most frequent indication in all first STEP patients (71%), while the indications for re-STEP were equally distributed between feeding intolerance and infections. Our experience with the STEP procedure also supports the statement that intestinal dysmotility with/without bacterial overgrowth or poorly correctable lactic acidosis are the most common indications for the lengthening procedure [27]. Nes et al. investigated factors associated with D-lactic acidosis in 46 children with IF and SBS. Fifty percent of the children with SBS had elevated D-lactate serum levels. A multivariable analysis in this trial identified midgut volvulus, a history of intestinal lengthening procedures, and an anion gap as significant independent risk factors in the development of D-lactic acidosis [43].

### 3.6. Increasing Intestinal Transit Time

**Figure 4:**
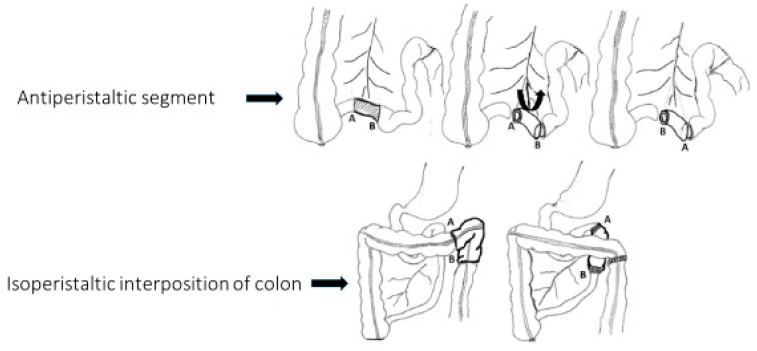
Antiperistaltic intestinal segment is created by reversal of gut segment that is located shortly before the ileocecal valve. Isoperistaltic interposition of the colon is performed with a segment of the left colon being introduced into the most proximal part of the small intestine.

Increasing the intestinal transit time in children with SBS allows sufficient time for digestion and absorption. Furthermore, prolonging the contact duration of intraluminal nutrients with intestinal mucosa stimulates intestinal adaptation and regrowth. Several surgical alternatives have been described to slow the intestinal transit time, including segmental reversal of the small bowel, artificial intestinal valve construction, and colonic interposition. Given the limited number of case series evaluating these surgical methods in both pediatric and adult patients, it remains challenging to identify the most effective conservative procedure for individuals with short bowel syndrome (SBS). An antiperistaltic intestinal segment is created by reversal of the gut segment (10 cm in adults and 3 cm in infants) that is located shortly before the ileocecal valve (ICV). This antiperistaltic segment acts as a physiological valve by causing retrograde peristalsis, serving as an effective brake to prolong the contact time of the chyme with the intestinal epithelium (Figure 4). Beyer-Berjot et al. have described their experience with segmental reversal of the small bowel in 38 adult patients with SBS. At the 5-year follow-up, 17 patients had been weaned from PN (45%), with a median weaning time of 14 months. In the remaining patients, the PN dependency had decreased from 7 to 4 days per week. Six patients had major complications and four patients required early reoperation [44]. Since the presence of an ileocecal valve (ICV) is reported by many investigators as an important predictive factor in achieving enteral autonomy, several methods of construction a non-refluxing valve to use as a substitute for the ICV in patients with SBS has been described [45].

Isoperistaltic interposition of the colon is performed with a 10–15 cm segment of the left colon, which is introduced into the most proximal part of the small intestine (Figure 4). This technique allows for a slow delivery time of nutrients to the small bowel without using any intestinal surface area. This technique has been described in adult patients with SBS and only a few clinical cases have been reported in children, with good outcomes in 50% of patients.

In the last four years, we found only one article discussing intestinal transposition, suggesting that these techniques have more historical than practical significance. H. Alhellani et al. recently described their results of skipped aganglionic lengthening transposition (SALT) in selected patients with intestinal failure secondary to total intestinal aganglionosis. In this technique, the interposition of three 5 cm pedicled isoperistaltic aganglionic ileal loops was performed between four normoganglionic jejunums (each 10 cm). This procedure resulted in a 36% increase in small bowel length. The authors conclude that SALT is effective for improving nutrient absorption in patients with total intestinal aganglionosis due to increasing the absorptive surface and prolonging the contact time between the enteric chyme and intestinal mucosa [46].

### 3.7. New Techniques and Combined Procedures

In the last decade, several new techniques for addressing a limited bowel length and less invasive modalities have emerged. Rafeeqi et al. performed spring-induced intestinal lengthening in adult and juvenile pigs. Springs were formed by the heat treatment of coiled nitinol filaments and were then compressed to 2 cm within a gelatin capsule; these were then coated with cellulose phthalate acetate. The encapsulated spring was inserted 10 cm proximal to the enterotomy. The authors have shown that spring segments lengthened the intestine, on average, by 86% in adult and 123% in juvenile pigs when compared to the initial length [47]. Distraction enterogenesis (through mechanotransduction) has recently been proposed to induce significant intestinal growth. Although this application has been limited to animals at this time, it has been demonstrated to lead to absolute growth in intestinal mass, and recent reports have described the use in this technique in clinical setting. Cunningham et al. describe a novel technique that was used to lengthen ileum in four children with ultrashort bowel syndrome and a preserved ileocecal valve. Following the laparoscopic lysis of adhesions, cecopexy is performed in the right-lower quadrant. A balloon catheter is inserted through a left flank stab incision and into the lumen of the remnant ileum, which is filled with radio-opaque contrast (to be visualized on subsequent radiographs), and fixed externally via an umbilical cord clamp followed by the application of increasing gentle tension distraction until the end of the catheter is reached or the tube is dislodged. After a median time of 132 days, intestinal continuity was restored. At the time of restoration, a median of 1.75 cm or 45% additional ileal length was achieved [48]. Valentini et al. designed a hybrid auxetic structure where the microorganism metabolic activity yields the formation of buckled/collapsed bubbles within gelling silicone cylinders that are applied to the longitudinal intestinal lengthening and tailoring procedure to promote enteral autonomy in short bowel syndrome in the porcine model. The authors believe that the presented material and analytical design synergistic approach offer a pioneering step for the clinical translation of hybrid auxetic materials [49]. Shun et al. describe a novel technique of double barrel enteroplasty (DBE) for autologous intestinal reconstruction in children with SBS and report their experience of 10 patients treated by this method of bowel lengthening. Five of the 10 patients achieved enteral autonomy (50%), while the remaining were on weaning PN. There were no serious complications in this cohort and no patients have required a liver and/or intestinal transplant. The authors conclude that double barrel enteroplasty has similar efficacy to STEP and LILT, but has fewer complications and is feasible and safe [50].

Gigola et al. presented their experience with combined procedures as the primary treatment in 21 children with SBS (preoperative median small bowel length of 20 cm) from two tertiary European Centers. Combined procedures were defined as more than one technique used on the same patient that combine lengthening, tailoring, and transit-time reducing procedures. The combined procedures were simultaneous in 15 patients and sequential in 6. Two patients achieved EA, and others followed a weaning home parenteral nutrition regimen with a median of four nights off. The authors concluded that combined gastrointestinal reconstructive surgery may be considered as a resource in intestinal rehabilitation units’ armamentarium [51].

### 3.8. AGIR in the Era of Teduglutide

In recent years, the use of glucagon-like peptide 2 (GLP-2) (teduglutide) to enhance remnant bowel adaptation has been suggested as a medical therapy for intestinal failure, with emerging evidence of its effectiveness and safety profile. Currently, teduglutide is approved for the treatment of SBS in children >1 year of age and has been associated with a decreased need for PN support (20%), improved enteral autonomy, and increased overall survival. In a pediatric study, 69% of patients receiving 0.05 mg/kg/day of teduglutide achieved the endpoint of a 20% or greater reduction in PN/IV at 24 weeks. This translated into a reduction of approximately 40% in PN/IV fluids and calories, and 10% of patients achieved enteral autonomy [52]. In accordance with another study, 76.9% and 82.1% of children with SBS who were treated with teduglutide reached a 20% or greater reduction in PN at 48 and 96 weeks, respectively, compared to 42.9% of non-treated patients. The cumulative proportion of patients in any TED group achieving enteral autonomy was 19.2% and 21.8% at 48 and 96 weeks, respectively, versus 14.3% and 14.3% of patients at weeks 48 and 96 in the non-treated group, respectively [53].

The impact of teduglutide on intestinal adaptation is suggested by the association of an increasing duration of exposure with the PN volume reduction. However, the impact of treatment with teduglutide on the need for surgery is unknown. In fact, the frequency of lengthening procedures in the management of pediatric SBS has decreased dramatically over the past decade. Moreover, the main indications for lengthening procedures have undergone significant changes. While the first report from the STEP Registry [22,39] described a impossibility of weaning off of PN as the main indication for STEP procedures, the last trials reported intestinal dysmotility and bacterial overgrowth as the main indications for AGIR [27,35]. Future studies should examine whether there are differences in the need for AGIR in pediatric patients with SBS related to positive or negative responses to teduglutide treatment.

### 3.9. Limitations

This review has several limitations. The heterogeneity in reporting by eligible studies on the outcomes and complications of AGIR makes it challenging to compare different procedures. Additionally, selection bias may be present in these studies, as different procedures may be performed based on specific anatomical characteristics. For instance, LILT is recommended when there is a sufficient length of dilated bowel with optimal intestinal blood supply, while STEP is suitable for short, unevenly dilated segments of small bowel, potentially with less optimal intestinal blood supply. Finally, small numbers of patients at individual centers, heterogeneous patient anatomy, and a lack of standardized definitions for SBS/IF and other important clinical outcomes make the comparison of results between institutions difficult, and more efforts are required to be further align the results across studies to advance the field through collaborative efforts.

### 3.10. Further Directions

Further research with pediatric patients with SBS-IF is necessary to identify the most effective bowel lengthening procedure for different patient profiles. This information will assist pediatric surgeons in selecting the appropriate procedure. In addition, clear recommendations could be made on the effectiveness of reSTEP after prior lengthening procedures. Future research is likely to focus upon the potential effect of the experience of the surgeon or institution with the surgical procedure on the outcome. Networking between centers and the recent publication of updated guidelines for the management of SBS should help standardize care between centers.

## 4. Conclusions

The primary goals of SBS therapy are the reduction or elimination of dependence on PN, optimization of enteral nutrition, and hydration, all of which substantially increase the probability of successful PN weaning. When enteral nutrient absorption falls to below one-third of the premorbid capacity and PN has failed to provide the daily requirement, autologous gastrointestinal reconstructive surgery is usually required. STEP remains the most popular surgical option in the management of children with SBS, especially in children with intestinal dysmotility, as it allows a decrease in PN dependency in most cases, with a weaning off of PN in half of the observed cases, and is characterized by low mortality. LILT is the second principal procedure to lengthen the native bowel in children with SBS. LILT was found to have an increase of 70% in small bowel length, equivalent to STEP, but a higher mortality. Over the past decade, several new techniques have been described as options for limited bowel length and the development of less invasive modalities.

## Figures and Tables

**Table 1:** The inclusion and exclusion criteria used according to the PICOS model.

PICOS Category	Inclusion Criteria	Exclusion Criteria
P (Population)	Children with SBS, who underwent AIRS.	Children with IF due to motility problems and children with ≥25% residual bowel length.
I (Intervention)	Full-text papers, including analytical cross sectional studies, prospective cohort studies, case control studies, longitudinal studies, randomized controlled trials, case series and retrospective cross-sectional studies.	Studies with incompatible virtual methods, studies whose primary focus was SBS without AGIR.
C (Comparators)	Studies comparing outcomes STEP vs. LILT vs. SILT or other comparisons between different surgical techniques.	Studies comparing surgical procedures in adult vs. children with SBS
O (Outcomes)	Survival and achievement of enteral autonomy (wean off PN). The rate of complications.	Incomplete results.
S (Study design)	Randomized and non-randomized controlled studies; longitudinal and transferal studies; review articles; studies written in English.	Duplicates; conference papers and abstracts; case reports; studies written in a language that was not English.

SBS—short bowel syndrome; IF—intestinal failure; AIRS—autologous intestinal reconstructive surgery; STEP—serial transverse enteroplasty; LILT—longitudinal intestinal lengthening technique, SILT—spiral intestinal lengthening and tailoring.

**Table 2:** Main outcomes of STEP procedure listed per type of intervention studied by authors.

Mortality	Decrease in PN Dependency (%)	Enteral Autonomy (%)	Increase in Bowel Length (%)	No Patients	Study
NR	19%	42%	50%	36	Dagorno et al. (France, 2024) [24]
5.5%	36%	32%	42–100%	308	Lauro et al. (review) (Italy, 2022) [25]
4%	NR	35	65	20	Cardoso Almeida et al. (UK, 2022) [26]
0	NR	73	70%	11	Eshel et al. (Israel, 2022) [27]
6%	NR	73	NR	16	Bueno et al. (Spain, 2022) [28]
7%	24 to 100%	45%	49.5%	377	Nagelkerke et al. (review) (The Netherlands/Finland, 2022) [14]
NR	81%	37.5%	60%	24	Lemoine et al. (USA, 2021) [29]
NR	50%	60%	25% (2STEP)	23	Mercer et al. (USA, 2021) [30]

STEP—serial transverse enteroplasty; PN—parenteral nutrition; NR—not reported.

**Table 3:** Main outcomes of LILT procedure listed per type of intervention studied by authors.

Mortality	% Decrease in PN Dependency	Enteral Autonomy (%)	Increase in Bowel Length (%)	No Patients	Study
NR	19%	42%	50%	36	Dagorno et al. (France, 2025) [24]
5.5%	36%	32%	42–100%	308	Lauro et al. (review) (Italy, 2022) [25]
4%	NR	44	70	9	Cardoso Almeida et al. (UK, 2023) [26]
22%	66%	52%	NR	324	Nagelkerke et al. (review) (2022) [14]

## Data Availability

The original contributions presented in the study are included in the article, further inquiries can be directed to the corresponding author.

## Abbreviations

AIRS	autologous intestinal reconstructive surgery
EA	enteral autonomy
IF	intestinal failure
LILT	longitudinal intestinal lengthening technique
PN	parenteral nutrition
SBS	short bowel syndrome
SILT	spiral intestinal lengthening and tailoring.
STEP	serial transverse enteroplasty
